# RELATIONSHIP BETWEEN SOCIAL PROBLEM-SOLVING SKILLS AND CAREGIVER BURDEN AMONG DEMENTIA CARE PARTNERS

**DOI:** 10.1093/geroni/igad104.2879

**Published:** 2023-12-21

**Authors:** Chung Lin (Novelle) Kew, Matthew Smith, Gang Han, Brittany Wright, Alexandra Holland, Alka Khera, Kristin Wilmoth, Shannon Juengst

**Affiliations:** Texas A&M University, College Station, Texas, United States; Texas A&M University, College Station, Texas, United States; Texas A&M University, College Station, Texas, United States; UT Southwestern Medical Center, Dallas, Texas, United States; UT Southwestern Medical Center, Dallas, Texas, United States; University of Texas Southwestern Medical Center, Dallas, Texas, United States; UT Southwestern, Dallas, Texas, United States; TIRR Memorial Hermann, Houston, Texas, United States

## Abstract

**Background:**

This study examined the extent to which social problem-solving abilities, care situations, and depressive symptomatology were associated with caregiver burden among care partners of adults with Alzheimer’s Disease and Related Dementias (ADRD).

**Methods:**

Baseline survey data were analyzed from care partners of ADRD who participated in a larger state-funded study. We used validated measures to assess social problem-solving abilities, care situation, positive aspects of caregiving, depressive symptomatology, and caregiver burden. Multivariate regression analysis with backward stepwise entry was used to identify factors associated with caregiver burden.

**Results:**

Care partners were, on average, age 60.4 (±12.4) and most were female (82.5%) and lived with their care recipient (69.1%). Care partners who reported high caregiver burden were more likely to be male (p< 0.0001) and have a positive problem-solving orientation (p=0.0002), but less likely to have a negative problem-solving orientation (p=0.0376). Higher caregiver burden was also associated with more resentment for caregiving role (p< 0.0001), more anger towards care recipients (p< 0.0001), more depressive symptomology (p< 0.0001), and higher positive aspects of caregiving (p=0.0010). Care partners who reported high caregiver burden also reported less social support (p=0.0076) and less family conflict (p=0.0159).

**Discussion:**

Findings suggest that factors thought to be protective against caregiver burden may not sufficiently reduce caregiver burden when considering their feelings of resentment and anger towards their care recipient, limited perceived social support, and high depressive symptomology. Interventions to reduce caregiver burden must consider care partners’ internal conflict as a caregiver and the relationship between the care partner and their care recipient.

